# Association of common WRAP 53 variant with ovarian cancer risk in the Polish population

**DOI:** 10.1007/s11033-012-2273-9

**Published:** 2012-11-29

**Authors:** Krzysztof Mędrek, Piotr Magnowski, Bartłomiej Masojć, Anita Chudecka-Głaz, Bogdan Torbe, Janusz Menkiszak, Marek Spaczyński, Jacek Gronwald, Jan Lubiński, Bohdan Górski

**Affiliations:** 1 Department of Genetics and Pathology, Pomeranian Medical University, Szczecin, Poland; 2 Department of Gynecological Surgery and Gynecological Oncology of Adults and Adolescents Pomeranian Medical University, Szczecin, Poland; 3 Department of Gynecological Oncology, Karol Marcinkowski University of Medical Sciences, Poznan, Poland; 4 West Pomeranian Center of Oncology, Szczecin, Poland

**Keywords:** WRAP53, TP53, Ovarian cancer, Genotyping, SNP, Polish population

## Abstract

Among many alterations within the TP53 gene the rs1042522 (C72G, p.Pro72Arg) has been associated with numerous cancers , however the results differ between populations for opposite Pro or Arg alleles. Similar thus inconclusive results are observed in ovarian cancer, which may suggest that the rs1042522 does not influence ovarian carcinogensis directly, but might be linked to another pathogenic alteration. WRAP53 which overlaps the TP53 is required to maintain normal levels of p53 upon DNA damage, but also when altered may independently increase the risk of cancer. To evaluate the association between three SNPs located in WRAP53–TP53 region: rs1042522, rs2287497, rs2287498 and ovarian cancer risk in Polish population we genotyped 626 cases and 1,045 healthy controls. Our results provide the evidence for an association between studied SNPs and a risk of invasive ovarian cancer in Poland. We found that CC homozygotes in rs1042522 were more frequent in cancers when compared to controls (OR = 1.46, *p* = 0.03). Similarly in WRAP53 both TT homozygotes in rs2287497 (OR = 1.95, *p* = 0.03) and AA homozygotes in rs2287498 (OR = 2.65, *p* = 0,01) were more frequent among cases than healthy individuals. There is also a suggestive evidence that specific homozygosity of studied SNPs in TP53–WRAP53 region is significantly overrepresented in ovarian cancer patients. In conclusion SNPs in WRAP53 (rs2287497 and rs2287498) have stronger association with an ovarian cancer risk than rs1042522 in TP53.

## Introduction

The TP53 is one of the most distinctive genes in carcinogenesis. Role of the p53 protein in regulation of cell cycle, cell growth and maintenance of genomic stability has been largely described. Numerous alterations in p53 lead to neoplasmatic transformation. A rare familial Li-Fraumeni Syndrome was associated with germline mutations in this gene. In families with Li-Fraumeni syndrome most commonly observed neoplasms are: soft tissue sarcomas, osteosarcomas, brain tumors, breast cancer, leukemias, and adrenocortical tumors, but not ovarian cancer [1] However, over 200 epidemiologic studies evaluated ovarian cancer risk associated with SNP variants in TP53. Among them the TP53 215 G>C (rs1042522, p.Pro72Arg) is one of the most commonly disputed. The results differ between populations with little or no evidence for the risk of cancer for opposite Pro or Arg alleles [2–8]. Similar thus inconclusive results are observed in ovarian cancer [9–11] and thereby reproduce previous findings. These equivocal data may suggest that the TP53 rs1042522 does not influence carcinogensis directly, but might be linked to another pathogenic alteration responsible for higher risk of ovarian cancer. One of the candidate gene is the WRAP53 (also denoted as WDR79 and TCAB1) located on chromosome 17p13 which partly overlaps the TP53 and interacts with the 5′UTR of p53. WRAP53 is required for control and induction of p53 upon DNA damage to maintain normal levels of p53 in the cell preventing its deprivation which may then lead to a cancer [12] The identification of Wrap53 as a regulator of p53 shows that dysfunction of Wrap53 itself may be a separate cause leading to a cancer [13, 14]Recent reports suggested that common variations in WRAP53 may independently increase risk of estrogen receptor negative breast cancers [15] and correlates with poor prognosis of head and neck cancer [16].

Aim of this study was the analysis of association between SNPs located in WRAP53–TP53 region: rs1042522, rs2287497, rs2287498 and ovarian cancer risk in the Polish population.

## Materials and methods

The studied group consisted of 626 ovarian cancer patients from the registry of International Hereditary Cancer Centre in Szczecin, Poland, who were diagnosed in years 2003–2009 in cooperating Oncology Centers in Szczecin and Poznań. All cases were histologically or cytologically confirmed. The control population consisted of 1045 healthy women who visited their family doctors in the area of Szczecin. Mean age and age structure did not differ significantly between cases and controls (age range 26–79 vs 27–79, mean age 55.5 vs 56 respectively).

The study protocol was approved by the Institutional Review Board of the Pomeranian Medical University, the approval was obtained from all cooperating institutions, and signed informed consent for genetic testing was obtained from all participants.

10 ml of peripheral blood samples for DNA extraction were acquired from cases and controls. The DNA extraction was performed in all individuals using the non-enzymatic method [17].

All samples were genotyped with use of Simple Probes on Roche LightCycler 480 in 384 well plates in 10 μL PCR reaction using 1:10 dilution of LightCycler 480 Genotyping Master, 0.5 μM of excess primer, 0.05 μM of limiting primer, 0.6 μM of probe and ~20 ng of DNA according to Roche Applied Sciences Genotyping Master Mix instructions.

All statistical calculations were performed using the common homozygous of each SNP as the reference value. Each of the SNPs was evaluated individually per genotype by calculating odds ratio (OR), 95 % confidence interval (95 % CI), *p* values using a chi^2^ test.

Genotype distributions of all three examined SNPs in control population were in accordance with Hardy–Weinberg equilibrium: rs1042522 (MAF = 0*.28, HWE* = 0.19*);* rs2287497 (MAF = *0.12, HWE* = 0.65*),* rs2287498 (MAF = 0.10*, HWE* = 0.57*).*

## Results and discussion

The distribution of the genotypes among ovarian cancer samples revealed an association of studied SNPs with ovarian cancer risk. Rs1042522 CC homozygotes were more frequent in cases when compared to controls (OR = 1.46) (Table 1). Similar association was observed for WRAP53 rs2287497 and rs2287498 where TT homozygotes and AA homozygotes, were more frequent among cases than controls (OR = 1.95, 2.65, respectively) (Table 1). All the results were statistically significant.

**Table 1:** Distribution of genotypes among ovarian cancers

Site		Cases		Controls		OR	*p*	CI 95 %	
		*n*	(%)	*n*	(%)				
rs10425225	CC	59	9.4	72	6.9	1.46	0.03	1.00	2.11
	CG	265	42.3	436	41.7	1.08	0.25	0.88	1.33
	GG	302	48.2	537	51.4	1.00	1.00		
rs2287497	TT	20	3.2	17	1.6	1.95	0.03	1.01	3.76
	TG	118	18.9	220	21.1	0.89	0.19	0.72	1.18
	CC	488	78.0	808	77.3	1.00	1.00		
rs2287498	AA	19	3.0	12	1.2	2.65	0.01	1.27	5.50
	GA	99	15.8	184	17.6	0.90	0.24	0.69	1.18
	GG	508	81.2	849	81.2	1.00	1.00		

A combined analysis of examined three SNPs showed an even stronger association with ovarian cancer risk for cases with three rare homozygotes CC + TT + AA versus wild type homozygotes GG + CC + GG (OR = 4.76 *p* = 0.0006 CI 95 % 1.84–-12.32). These calculations were statistically significant and included Bonferroni correction. However, detailed studies around this region will be needed to clarify the observed association of that specific homozygosity in other populations.

The ambiguous associations found for rs1042522 with little or no evidence for the risk of ovarian cancer were previously described in different populations, similarly neither TP53 germline mutations nor Li-Fraumeni syndrome was proven to be associated with an increased risk of ovarian cancer. Hence the hypothesis that an evaluation of SNPs located in close proximity to TP53 may reveal that rs1042522 is just a marker of other pathogenic alleles. WRAP53 is one of the candidate flanking genes to TP53 located on antisense strand to TP53. An analysis of the literature revealed studies involving genotyping of 23 SNPs in the TP53 region including some variants outside the TP53 coding sequence, among them SNPs located in fact within WRAP53. Previously published multicenter pooled data suggested an association between rs2287497 and rs2287498 with serous invasive ovarian cancers, but the results were inconsistent between analyzed populations [10, 11]. rs2287498 is a synonymous amino acid change in exon 2 (F150F) of WRAP53 gene, expected to affect function at a splice site, rs2287497 is an intronic change, however supportive of an association [11]. The study of Schildkraut included a small number of Polish ovarian cancer patients, however narrowed to serous ovarian cancer cases only, because of the a priori belief that TP53 variants might be more closely related to serous cancers [10]. Schildkraut findings showed a trend consistent with our results, but due to relatively smaller groups (118 ovarian cancers vs. 618 controls) the scores were not statistically significant leaving the case open.

Our results provide the evidence for an association between SNPs both in TP53 and WRAP53 and a risk of invasive ovarian cancer in Poland. Our results might be a suggestive evidence that specific homozygosity of several SNPs in TP53—WRAP53 region is significantly overrepresented in ovarian cancer patients. The observations suggest a stronger association of both rs2287497 and rs2287498 (WRAP53 variants) than of rs1042522 (TP53 variant) and lead to a conclusion that SNPs in WRAP53 is more strongly associated with an ovarian cancer risk than rs1042522 in TP53. It is possible that other variants of WRAP53, not selected for the evaluation, are also related to the disease.
